# Flexible Adaptive Paradigms for fMRI Using a Novel Software Package ‘Brain Analysis in Real-Time’ (BART)

**DOI:** 10.1371/journal.pone.0118890

**Published:** 2015-04-02

**Authors:** Lydia Hellrung, Maurice Hollmann, Oliver Zscheyge, Torsten Schlumm, Christian Kalberlah, Elisabeth Roggenhofer, Hadas Okon-Singer, Arno Villringer, Annette Horstmann

**Affiliations:** 1 Department of Neurology, Max Planck Institute for Human Cognitive and Brain Sciences, Leipzig,Germany; 2 Leipzig University Medical Center, Leipzig, Germany; 3 NMR Unit, Max Planck Institute for Human Cognitive and Brain Sciences, Leipzig, Germany; 4 Leipzig University Medical Center, Leipzig, Germany; 5 Leipzig University Medical Center, IFB Adiposity Diseases, Leipzig, Germany; 6 Clinic for Cognitive Neurology, University Hospital, Leipzig, Germany; 7 Department of Psychology, University of Haifa, Haifa, Israel; 8 Mind and Brain Institute, Berlin School of Mind and Brain, Humboldt-University and Charite, Berlin, Germany; University Medical Center Groningen UMCG, NETHERLANDS

## Abstract

In this work we present a new open source software package offering a unified framework for the real-time adaptation of fMRI stimulation procedures. The software provides a straightforward setup and highly flexible approach to adapt fMRI paradigms while the experiment is running. The general framework comprises the inclusion of parameters from subject’s compliance, such as directing gaze to visually presented stimuli and physiological fluctuations, like blood pressure or pulse. Additionally, this approach yields possibilities to investigate complex scientific questions, for example the influence of EEG rhythms or fMRI signals results themselves. To prove the concept of this approach, we used our software in a usability example for an fMRI experiment where the presentation of emotional pictures was dependent on the subject’s gaze position. This can have a significant impact on the results. So far, if this is taken into account during fMRI data analysis, it is commonly done by the *post-hoc* removal of erroneous trials. Here, we propose an *a priori* adaptation of the paradigm during the experiment’s runtime. Our fMRI findings clearly show the benefits of an adapted paradigm in terms of statistical power and higher effect sizes in emotion-related brain regions. This can be of special interest for all experiments with low statistical power due to a limited number of subjects, a limited amount of time, costs or available data to analyze, as is the case with real-time fMRI.

## Introduction

Brain responses obtained from fMRI time series identify brain activation involved in a given task or in response to certain stimuli. But these signal changes are most likely also influenced by task independent variables in addition to task specific effects. Firstly, it is known that fMRI results are heavily influenced by the subject’s compliance to instructions, like directing gaze and attention to visually presented stimuli [1,2]. Secondly, brain responses are related to physiological fluctuations [3–5] and experimental stimuli are differently perceived and processed over the course of such fluctuations [6]. In this context various questions arose repeatedly:

1. How do physiological fluctuations, such as pulse, heart rate variability or blood pressure, and a subject’s compliance influence the perception and processing of different kinds of stimuli?
2. How do we make sure that subjects are in a comparable state (e.g., relaxed, stressed, or anxious) for the application of a state-specific stimulation?
3. How do we make sure subjects attend to the presented stimuli?
4. How do brain states in specific areas influence the perception and processing of stimuli?

To answer these questions, a simultaneous acquisition of brain signals and physiological parameters is indispensable. When they are available, the standard approach is to include these parameters in the *post-hoc* analysis of the data to correct for confounds of physiological fluctuations over the course of an experiment. This is performed by the exclusion of trials or by correction algorithms [7,8]. This has two major disadvantages: First, the *post-hoc* exclusion of trials from the paradigm or even the exclusion of subjects from the study inherently involves a waste of time, money, and effort running useless trials. Second, *post-hoc* correction algorithms are not perfect and usually lead to signal reduction compared to the original measurement.

An alternative approach would be to adapt experimental stimuli in real-time as a function of information from different modalities. Instead of relying solely on data analysis, it allows to present trials only when objective criteria are met. For example, by integrating an eye tracker, the criteria for stimuli presentation can be certain parameters, such as subject’s gaze position and eye movement velocity. This *a priori* approach maximizes the efficacy of data collection. In other words, if stimuli are only presented when external criteria are met (e.g., the subject’s gaze position or resting pulse), all the trials from the paradigm included in the subsequent data analysis will be ‘valid’ in the given context.

As proposed by Wilms and colleagues, adaptive paradigms can make for more realistic experiments. Especially in the context of social cognitive and affective neuroscience this approach can be seen as a unique tool for ‘online’ interaction with subjects in an MR environment [9].

Beyond external parameters, adaptive paradigms are also required when the brain’s variability itself is to be explored. In such cases the stimulation schedule could be adapted according to the current brain states as assessed with other measures (e.g., EEG or the fMRI signal itself). In a single use setup, Becker and colleagues utilized this idea and explored the influence of EEG alpha power on visually evoked responses in fMRI. To be able to address this question they adapted the timing of the visual stimulation to the instantaneously processed EEG signals in a combined EEG-fMRI setup [11]. Recently, this approach was realized with the fluctuations in the fMRI signal itself by Yoo et al. [12]. In this study the stimuli were only presented when fMRI fluctuations of the target area were in a predefined ‘good’ or ‘bad’ state (high or low amplitude of the BOLD signal). The study resulted in an improved behavioral performance in the memorization of the stimuli when adapting picture presentation to the ‘good’ state of fMRI fluctuations.

For the purpose of adaptive paradigms, the stimulation schedule has to be changed in real-time with respect to these parameters. These so-called *adaptive paradigms* have to meet several challenges:

1. Inclusion or exclusion criteria defining the validity of physiological and behavioral parameters have to be defined *a priori*.
2. Physiological and behavioral parameters have to be recorded in an MR environment and transferred to the stimulus system controlling the experiment during runtime.
3. Adaptation of the stimulation has to be performed promptly and correctly while the experiment is running.

The implementation of an adaptive stimulus presentation is technically feasible with available software packages, for example with Presentation (www.neurobs.com), as done by Wilms et al. [9], Cogent (www.vislab.ucl.ac.uk/cogent_2000.php), CIGAL (www.nitrc.org) by Voyvodic et al. [10] or Paradigm (www.paradigmexperiments.com). But the implementation of an adaptation must be coded in the software’s native language or a provided scripting language and is limited by the manufacturer’s driver support.

Unfortunately, each of the presented setups only covers a single case of experimental use. To date, no integrated framework exists to represent an adaptive stimulation in general. In other words, available software packages lack a standardized description of an adaptive behavior that is straightforward to setup and reuse with different input signals. Therefore, this need for a general framework for the adaptation of fMRI paradigms motivated us to develop a new, combined software package.

**Fig 1:**
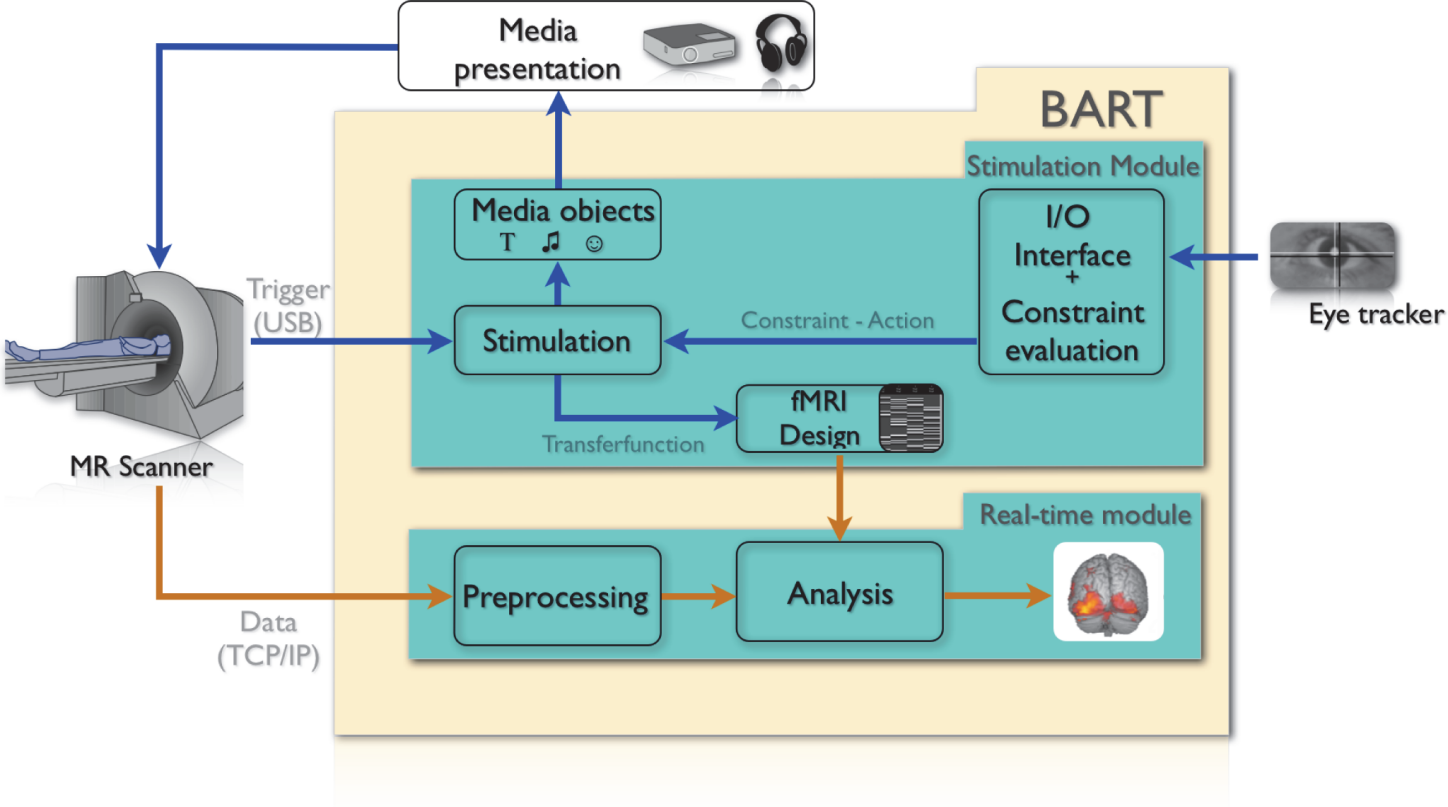
Schematic overview of the software package BART. Shown are the modules each running as a separate and independent process, responsible for the inclusion of behavioral/physiological parameters, the adaptation of the stimulation, design generation (blue arrows), and fMRI data processing (orange arrows). In the context of the presented study, we used an eye tracker to adapt visually presented emotional pictures according to subjects’ fixation on the center of the screen. The plugin mechanism also allows for straightforward and flexible extension to other devices, like ECG, pulse or EEG.

This report describes our approach to the implementation of adaptive paradigms in two steps. Firstly, we introduce a new open source and publicly available software tool called ‘Brain Analysis in Real-Time’ (BART, https://github.com/bart-group/BART), which considers all the challenges as described above (see Fig. 1 for an overview). BART is a unified software framework that integrates an adaptive stimulation environment, which is the focus of the current work, and real-time fMRI analysis in a single computer setup. Secondly, we demonstrate the benefits of adaptive paradigms as applied by BART with a usability example. In fMRI-based emotion research, it is general practice to use paradigms consisting of pictures with negative, neutral or positive contents with different valence and arousal ratings from the international affective picture set (IAPS) [14]. The basic idea of the current study is to analyze data from an eye tracking system in real-time to track the subject’s gaze and to adapt stimulation to the effect that IAPS pictures are presented only when the subject fixated on the center of the screen. This approach is expected to result in enhanced neural activation in regions implicated in visual perception and emotion processing, when compared to conventional stimulation uncontrolled for gaze direction. To demonstrate the advantages of this approach, we ran a study comparing in a within-subject design between conventional and adaptive presentation in a group of healthy subjects.

## Methods

### Software development

The software package BART is developed in an object-oriented approach using the programming languages Objective-C and C++. It consists of logical units implemented as independent modules separated into two main components: the ‘*stimulation’* module and the ‘*real-time*’ module. The ‘*stimulation*’ module, which is the focus of the current paper, allows one to adapt the stimulation by an abstract description of the adaptation process. This is done in concert with a plugin mechanism that allows the on-demand integration of various external devices, like EEG, ECG, or eye tracker (see Fig. 1). This generalized approach guarantees maximum flexibility according to each experimenter’s requirements because any external or internal variables can be used to control the stimulation in a standardized manner. The stimulation module is a core component of the software package. There are no additional changes necessary for a new adaptive paradigm. All the specific knowledge regarding the adaptation parameters is implemented into the external plugins. Here, we present the plugin for an eye-tracking device, but with this plugin mechanism it can be easily extended to further devices. Furthermore, over the course of an experiment, the dynamically created design can be transferred to a real-time general linear model (GLM) analysis to adapt the design matrix used for online statistics.

For the implementation of adaptive paradigms based on flexible external parameters, a complete configuration of the experiment and an abstract description of the adaptation process are necessary. For this purpose, BART’s configuration is based on the Experiment Description Language (EDL) developed by Hollmann et al. [15]. This language is an XML-based structured description of all parameters relevant for real-time data acquisition, statistical data analysis, stimulus presentation, and activation processing (e.g., feedback generation). It also comprises a knowledge base of rules describing the dependencies between these parameters. This configuration helps experimenters to setup a valid design before scanning. For the configuration language EDL a platform independent editor is available, which was implemented by Oliver Zscheyge [22] (https://github.com/ooz/EDLed). This editor simplifies the generation of configuration files and keeps track of the compliance specified by the contained knowledge base rules.

All parts of the fMRI paradigm are fully defined by the EDL-description of the experiment. The paradigm definition mainly consists of a list of all ‘*media objects*’, which are the stimuli themselves (text, picture, or audio file), and a ‘*timetable*’ containing the schedule for the presentation of the ‘*media objects*’. These two components are essential for realizing conventional fMRI paradigms.

For the purpose of flexible adaptive paradigms, we extended the language EDL by five new constructs, which describe the essential parts of an adaptation in an abstract manner (see Textbox 1 for a concrete example):

1. ‘*Constraints*’: logical constructs describing parameter dependencies for media objects
2. ‘*System variables’*: unique definitions of external devices and available parameters
3. ‘*Stimulus actions’*: actions to be done when constraints are fulfilled or not
4. ‘*Transfer functions*’: description of how an adapted trial has to be transferred to the design matrix for the data analysis
5. ‘*Dynamic Time-Based Regressor*’: regressor for the design matrix, which receives its data entries during runtime based on the adapted trials

For an adaptive paradigm the presentation of a trial (containing a ‘*media object*’) depends on the evaluation of a ‘*constraint*’. Based on this evaluation, a ‘*stimulus action*’ is executed. So far, there are three possible ‘*stimulus actions’*: (1) a stimulus can be replaced by another one, (2) a new trial can be created and, optionally, all following events are shifted in time according to the duration of the new trial or, (3) presentation parameters (e.g., picture position on screen) of a stimulus can be adapted. In addition to the adaptation process, the updated schedule of the stimulus presentation has to be transferred into the experiment’s design matrix used for the data analysis. Therefore, the ‘*transfer functions*’ define the trial onset, duration, and a unique assignment to a regressor for the data description.

### EDL-Configuration code example for the proof-of-concept study

# This is an example of a constraint that describes uniquely the eye tracker as the signal source. This ID will identify the external plugin, which deals with the eye tracking data, while loading.

<constraints>

<systemVariables>

<systemVariable source = "de.mpg.de.ASLEyetracker" systemVariableID = "sV1" systemVariableName = "eyePosIsFixated"/>

<systemVariable source = "de.mpg.de.ASLEyetracker" systemVariableID = "sV2" systemVariableName = "eyePosX"/>

<systemVariable source = "de.mpg.de.ASLEyetracker" systemVariableID = "sV3" systemVariableName = "eyePosY"/>

</systemVariables>

<constraint constraintID = "co1">

# The list of conditions which boolean result is evaluated by the external plugin during runtime.

<conditions>

<condition systemVariableRef = "sV1"/>

</conditions>

# This describes the action if the boolean result value is, false’.

<stimulusActions_else>

<stimulusAction>

# Here: the action inserts an event that displays a fixation cross for 8 ms and shift in time all following events.

<insertNewStimulusEvent duration = "8" mediaObjectRef = "FIXCROSS" shiftFollowingStimulusEvents = "true"/>

</stimulusAction>

</stimulusActions_else>

</constraint>

</constraints>

# This is an example of a stimulus that uses the constraint from above. Its parameters are transferred into the design matrix regressor, REG_NEGDYN’ after it has been displayed.

<mediaObject moID = "PicID1" name = "1019" type = "IMAGE" useConstraint = "co1" >

<contentImage>

<imageFile>pics/1019.jpg</imageFile>

<posX>0</posX>

<posY>0</posY>

</contentImage>

<regressorAssignment assignToRegressor = "REG_NEGDYN" useTransferFunction = "tranF1"/>

</mediaObject>

# This is an example for a concrete transfer function that transforms a stimulus into an event of the design matrix.

<transferFunctions>

<transferFunction transferFunctionID = "tranF1" timeOffset = "0" durationScaleFactor = "0" parametricScaleFactor = "1"/>

</transferFunctions>

# This is an example of a design matrix definition with the two regressors “REG_NEGDYN” and “REG_NEUTDYN”.

<paradigm ignoreScansAtStart = "20">

<dynamicDesignStruct maxLength = "4000000">

<dynamicTimeBasedRegressor length = "4000000" name = "neg_dynamic" regressorID = "REG_NEGDYN" scaleHeightToZeroMean = "false" useRefFct = "gloverKernel" useRefFctFirstDerivative = "false" useRefFctSecondDerivative = "false"/>

<dynamicTimeBasedRegressor length = "4000000" name = "neut_dynamic" regressorID = "REG_NEUTDYN" scaleHeightToZeroMean = "false" useRefFct = "gloverKernel" useRefFctFirstDerivative = "false” useRefFctSecondDerivative = "false"/>

</dynamicDesignStruct>

</paradigm>

### Benchmark testing

To test the performance of the software’s stimulation module under benchmark conditions, all processing units of the Mac Pro hardware (overall 16 processing units due to hyperthreading ‘Nehalem- microarchitecture’) were kept at maximum load with benchmark software tools (CineBench, http://www.maxon.net/; SystemLoad. http://www.bresink.de/osx/SystemLoad-de.html) while BART was running. This was tested for the standard and adaptive execution of the software. The latter case added 200 new events to the experimental schedule at the same time. In the high load condition 16 parallel threads were running when the adaptation took place.

Additionally, the timings for the evaluation of the constraints and processing of the actions, here the analysis of the eye tracker data, and shifting of trials have been tested under CPU loading conditions. Therefore, internal time measurements have been checked over 20 runs with 120 evaluations each for the adaptation. Updating the design matrix was averaged over 20 runs with 240 updates each. To verify the functionality of all processing steps unit tests were implemented in an additional module, which is available with the software package. In such tests, expected results of examples for all operations by the software are separately implemented and tested against the results of the software itself.

### Adaptive fMRI experiment with emotional IAPS pictures

**Fig 2:**
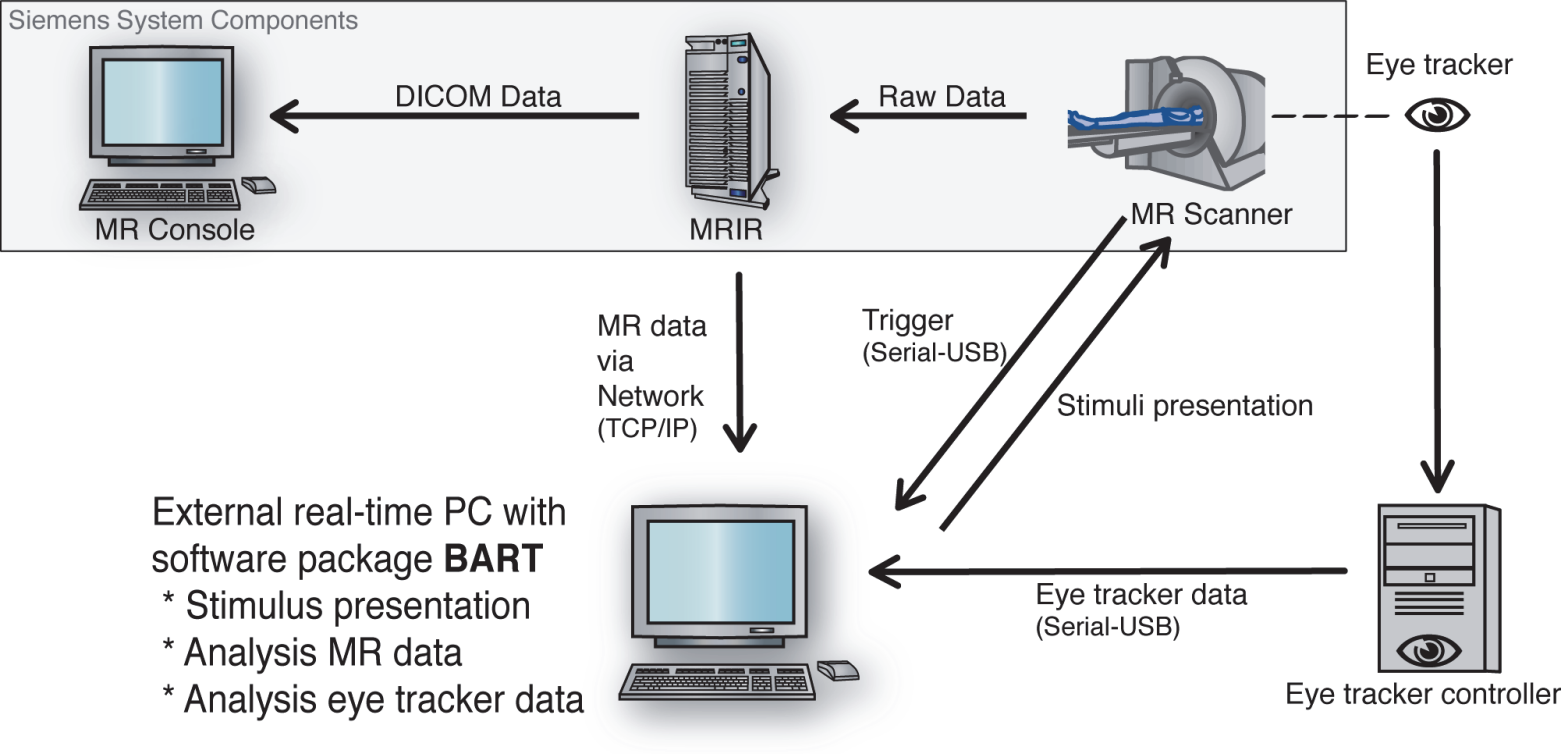
The experimental setup. A single external computer equipped with BART is connected with the internal Siemens reconstruction computer. This runs a custom-made software tool that sends MR data via network connection directly to the external instance. Additionally, eye tracker data are sent to this computer. They are computed in parallel to MRI data, the stimulus presentation, and the paradigm adaptation.

With the previously described abstract concept, we set up our first concrete fMRI experiment with an adapted paradigm containing constraints regarding the subject’s fixation state. This means pictures are only presented when the subject fixates the center of the screen. Otherwise, the picture onset is shifted forward in time until a fixation is recorded. The software adapts the timing of both the current stimulus onset and all following trials. The technical setup for the fMRI experiment, as shown in Fig. 2, consists of three components: (1) MR scanner; (2) Eye tracker; (3) External PC equipped with BART.

### Data acquisition

Functional images were acquired on a 3T whole body scanner with standard 12-channel head coil (Siemens MAGNETOM Trio, Tim System, Siemens, Erlangen, Germany). Based on the recommendation by Robinson and colleagues [16] for echo-planar-imaging (EPI)-protocols to acquire more reliable signals from limbic regions (e.g., the amygdala) of the brain, we used an EPI-sequence with the following parameters: TR = 2 s, TE = 28 ms, matrix size = 64 x 64 voxel, bandwidth = 1954 Hz, flip angle = 90°. Thirty-four axial slices (voxel size = 3 x 3 x 2.5 mm^3^, gap = 0.25 mm) aligned in AC/PC + 5° were acquired. In order to minimize head movements an additional foam cushion was used to immobilize the subject’s head.

Simultaneously, eye movements were recorded with an eye tracking system (ASL EyeTrac 6 Long Range Optics, Applied Science Laboratories, Bedford, USA). Eye movements were monitored using an infrared camera pointing at the subject’s head from outside the bore of the scanner. IAPS pictures were presented on a screen at the rear end of the scanner bore via a mirror mounted on the head coil above the subject’s head. This mirror was also used to direct the infrared light from the eye tracking system into a subject’s eye, thus measuring pupil diameter and corneal reflex. This data was sent to the controller unit of the eye tracking system outside the MR scanner room, which itself was connected to the external computer equipped with BART.

This external computer controlled the stimulation procedure utilizing the recorded eye tracker data and adapted the paradigm when necessary. The stimulation procedure in BART ran in synchronization with the repetition time of the MR scanner (by an optical trigger; here every 2 s).

### Subjects

Twenty-one healthy subjects (male 13; mean age 26.5 ± 4.3 years) with normal vision (no glasses, no contact lenses) participated in this study. All subjects gave written informed consent. Due to the use of highly negative pictures, the subjects were familiarized with several examples of the pictures prior to the experiment, in addition to the written instructions. The subjects were explicitly asked if they agreed to being exposed to such images. The local ethics committee at the Medical Faculty of the University of Leipzig approved the study in accordance with the Human Subjects Guidelines of the Declaration of Helsinki. Four subjects were excluded from the analysis because of incomplete scanning sessions due to head movement-based loss of the eye tracker signal.

Post-hoc, subjects were divided by median split into ‘Low-Compliant’ (n = 8) and ‘High-Compliant’ (n = 9) groups according to their fixation performance across all non-adapted picture presentations.

### Stimuli

**Fig 3:**
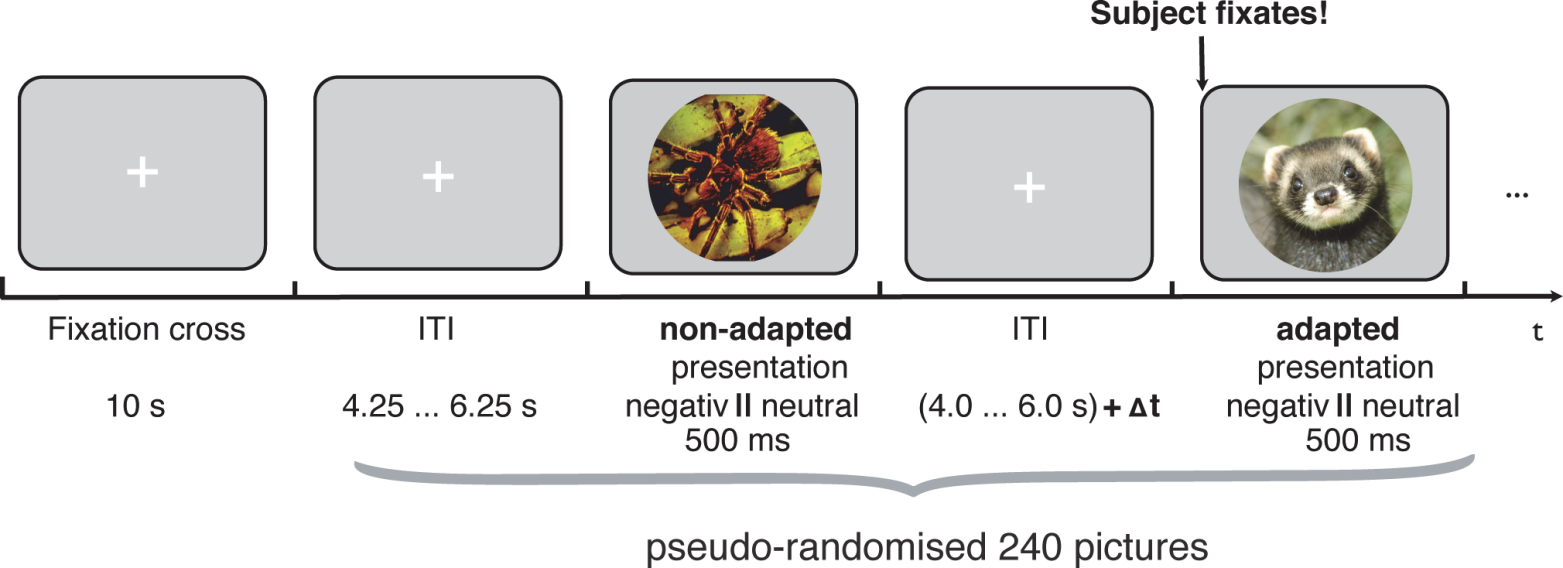
Experimental paradigm. The experiment’s paradigm consisted of 60 negative and 60 neutral IAPS pictures, each presented in adapted and non-adapted manner over the course of the experiment. Each picture was presented for 500 ms with a fixation cross in between. In the adapted condition the picture presentation was shifted in time until subjects directed their gaze position toward the center of the screen and hence fixated the center of the subsequently presented picture. The main purpose of the study was to investigate the benefits of this trial adaptation.

The paradigm consisted of 60 negative and 60 neutral IAPS pictures [14]. In order to control for complexity and eye movements during picture presentation, they were modified as follows: A circular section around the emotion-defining part of the presented scenario was chosen and put on a gray background (see examples in Fig. 3). The modifications of the pictures were validated in another study with 40 subjects rating the modified pictures according to valence and arousal. The ratings of the modified pictures matched those obtained for the original pictures (all Pearson correlation: valence r = 0.99; arousal 0.95; Hellrung et al., unpublished data).

Negative and neutral pictures were matched with regard to color, contrast, and illumination. All pictures were presented in an intermixed, pseudo-randomized order of negative/neutral pictures and adapted/non-adapted condition. This means that each picture was presented twice over the course of the experiment. To take into account the effect of the first reaction to a negative picture, they were matched according to valence ratings for their first presentation, either in the adapted or non-adapted condition. Therefore, 1000 picture randomizations were generated and the mean of the valence ratings of the current picture division was calculated for each randomization. Out of those, an individual randomization was chosen for each subject with a minimal difference in valence rating points for adapted vs. non-adapted negative pictures (criteria: mean less than 0.01 difference in rating points and standard deviation less than 0.1; range of rating points for valence and arousal was from 1 to 10).

### Paradigm

All pictures were presented at the center of the screen, for 500 ms with an inter-trial interval (ITI) ranging from 4 to 6.25 s (in steps of 1 s). During the experiment, for each adapted trial the stimulation environment of our software package BART checked the subject’s gaze direction, and, if necessary, shifted this trial forward in time until the subject fixated the center of the screen. Fig. 3 shows a schematic overview of the paradigm. Subjects were instructed to respond via a button press using the index and middle finger if they recognized a negative or neutral image on the screen (without an instruction to answer as fast as possible). After the fMRI scanning session, subjects rated the modified IAPS pictures according to valence and arousal with the Self Assessment Manikin (SAM) [18] in a computer experiment.

#### Online eye tracking data

For the adaptation process, the eye tracking data was analyzed in real-time via a plugin for BART during the experiment. This plugin provides an algorithm to identify the gaze position according to a definition by Salvucci and Goldberg. They describe a Dispersion-Threshold Identification for fixations with two parameters [17]. In the context of the present study, these were defined with a 200-ms duration threshold and a visual angle of 1° as dispersion threshold. Additionally, the gaze position was accepted as valid if the visual angle was within a 2° visual angle of the center of the screen. This criterion was chosen because of the modified circular pictures, which had a size of approximately 3° showing the most relevant content near the center of the screen.

### Data analysis

In the offline fMRI data analysis, pre- and post-processing of data was performed using the software package SPM8 (http://www.fil.ion.ucl.ac.uk/spm). All images were corrected for local magnetic field inhomogeneity, slice timing, and head movement. They were normalized into MNI (Montreal Neurological Institute, [19]) template space, smoothed with 8-mm FWHM (full-width at half-maximum) Gaussian kernel, and a high pass filter with a cutoff frequency of 128 Hz was applied.

For an intra-individual comparison of the effect of the stimuli, we used a GLM analysis as implemented in SPM8 [20]. On the single-subject level the GLM contained 4 regressors for each condition (‘negative-non-adapted’ (NGNA), ‘negative- adapted’ (NGA), ‘neutral-non-adapted’ (NTNA) and ‘neutral-adapted’ (NTA)). Additionally, the realignment parameters were included as nuisance covariates in the design matrix.

For the second level analysis, a flexible factorial design was used. A threshold of p < 0.001 uncorrected was accepted as significant. The main contrasts of interest have been: (1) the main effect of emotion ([NGA + NGNA]–[NTA + NTNA]) for all subjects, (2) the main effect of adaption ([NGA + NTA]–[NGNA + NTNA]) for High- and Low-Compliant separately and (3) if a difference is left between these two groups for the adapted trials ([NGA + NTA]*High-Compliant—[NGA + NTA]*Low-Compliant). For the last mentioned whole-brain comparison of non-adapted trials a two-sample t-test as implemented in SPM8 was used (p < 0.001).

To illustrate the influence of the adaptation on the effect sizes of each regressor, we used the rfxplot toolbox [21] in SPM8 for brain regions identified with whole-brain GLM statistics. Therefore, the identified MNI coordinates of the main emotional effect for every subject were used to extract the average signal across all voxels in a 3-mm sphere. Afterwards the effects were split into High- and Low-Compliant and a one-way ANOVA was applied to test for differences in the mean value of the effect sizes between the two groups.

#### Offline eye tracking data analysis

The same algorithm as described for the online analysis was implemented in the script language Perl for a post-hoc analysis of the trials. Therewith, a subject’s performance regarding their fixations for the non-adapted picture presentations is quantified, and the online adaptation is validated.

## Results

### Software package BART

Our software package BART is currently installed and being tested at two MRI facilities (Siemens TIM TRIO, Siemens Verio). It runs on Mac Pro hardware (2 Quadcore processors, 8 GB RAM with Mac OS X 10.7/10.8) and is setup as shown in Fig. 2.

The stimulation procedure is working reliably and runs in parallel to the data import and processing. The correctness of the adaptation is validated by a *post-hoc* fixation analysis for all adapted trials. The performance of this software module has been tested under the described benchmark conditions (see section 2.2). For both standard cases—no additional CPU load and no adaptation—the delay time between the event release and the finished drawing on the screen was always less than 2 ms. In the high load condition 16 parallel threads were running when the adaptation took place. Even under this extreme computational load, only minor delays of less than 7 ms were detected.

The evaluation of the constraints and processing of the eye tracker data and shifting of trials was finished in less than 1 ms. To be able to analyze the data immediately it is essential to update the design matrix accordingly during the runtime. Updating the design matrix took 15 ms on average (± 3.5 ms).

### Behavioral results

All subjects showed normal behavior regarding categorization of the IAPS pictures. The picture ratings according to valence and arousal strongly correlated with the results from the validation population (Pearson correlation coefficient for valence: r = 0.98; p < 0.001 and arousal: r = 0.96; p < 0.001).

**Fig 4:**
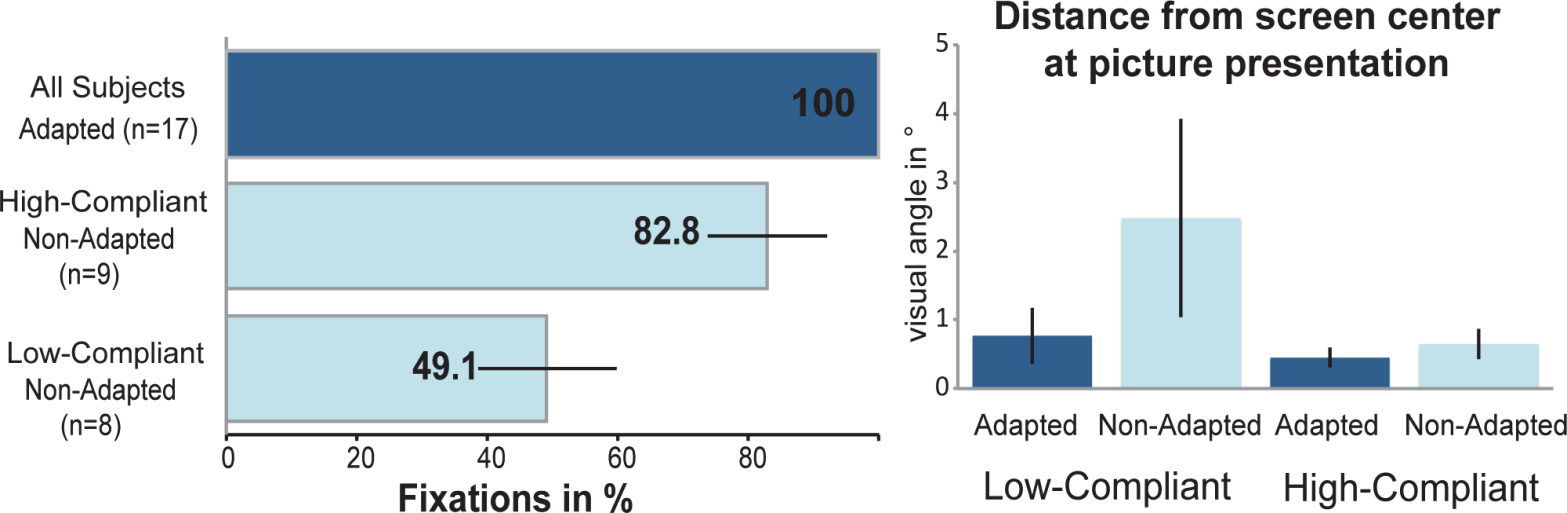
Behavioral results. The post-hoc analysis of eye tracking data shows the correct functionality of the software by 100% fixations for all subjects in the adapted condition. In addition, it shows difference in fixation compliance and gaze distance from the center of the screen for the non-adapted picture presentation. Due to the bimodal distribution of this parameter, subjects were divided into subgroups.

Offline, we examined the gaze position at the beginning of each picture presentation. For the non-adapted trials, these show that the overall fixation compliance was 67% (± 22%) for all subjects. The results have shown a bimodal distribution of this parameter. According to this, subjects were divided into two groups (‘High-Compliant’ and ‘Low-Compliant’) by a median split on fixation compliance. The High-Compliant group fixated in 83% of the non-adapted trials (± 6.2%), whereas the Low-Compliant group had a fixation rate of only 49% (± 20%). Additionally, we examined the gaze position at the beginning of stimulus presentation. In a descriptive manner, the results illustrate that in the non-adapted condition eye position of the Low-Compliant group does not coincide with the center of the screen in contrast to the High-Compliant group. There is no difference for the adapted picture presentation. Fig. 4 illustrates the behavioral results for fixation success and describes a difference for the distances between gaze position and screen center between the two groups. Due to the functionality of our software all trials for all subjects are valid in the adapted presentation condition, i.e. eye position deviates from screen center by less than 1° visual angle.

Behaviorally, we found a significant increase in the accuracy to distinguish neutral and negative pictures for the adapted presentation in the Low-Compliant group (adapted 92% ± 2.6%; non-adapted 88% ± 3.5%; p < 0.04; no difference for High-Compliant).

### FMRI experiment

**Fig 5:**
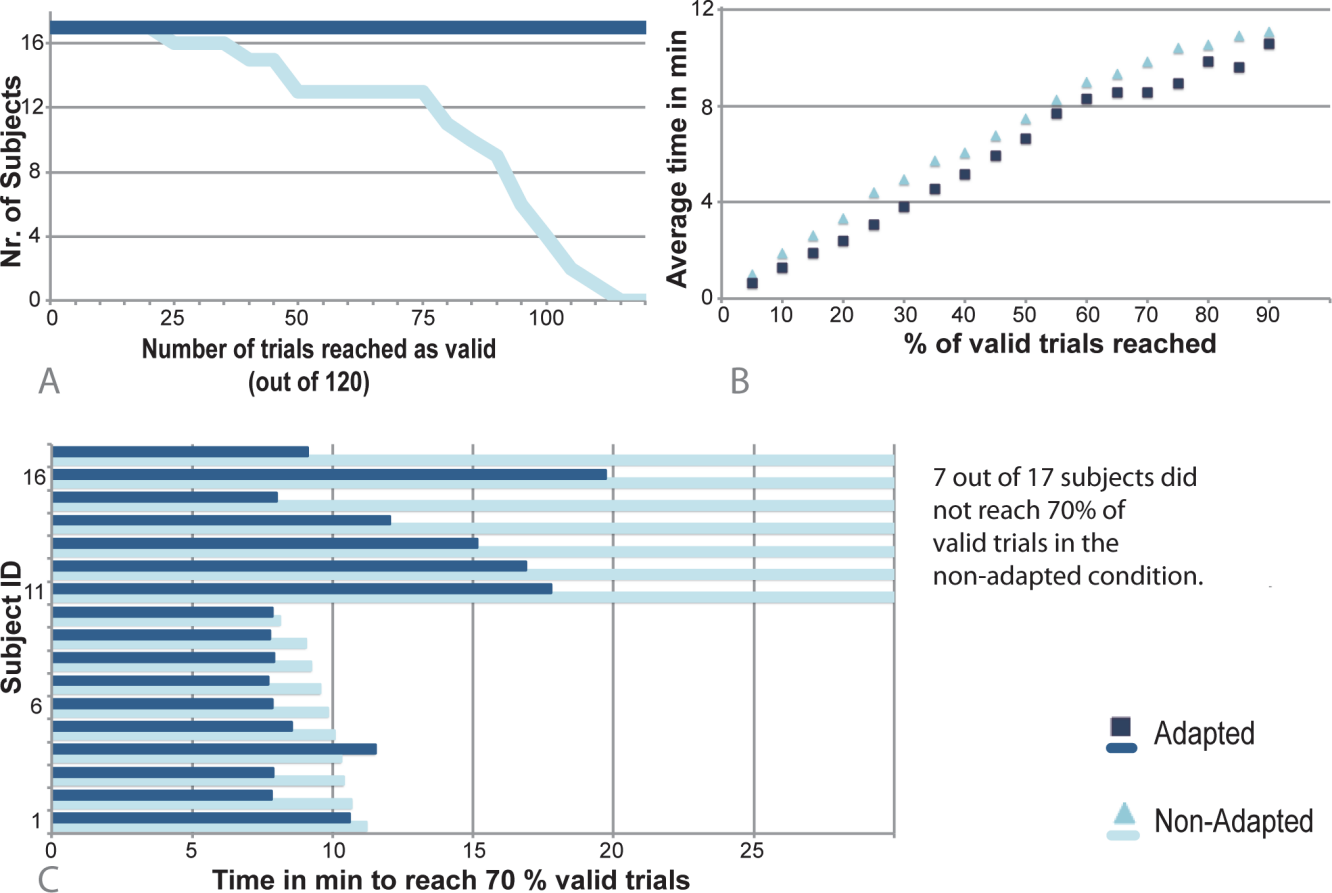
Comparison of experiment timing adapted and non-adapted condition. Individual rates of the amount of trials reached as valid regarding the fixation compliance (A); Overall comparison of time that was needed to reach a certain amount of valid trials. For 95% and 100% valid trials none of the subjects reached these amounts in the non-adapted manner, therefore comparison is not shown (B); Exemplarily, the individual results for this with the threshold of 70% valid trials. 7 out of 17 subjects did not reach this amount of valid trials in the non-adapted condition. 9 out of the 10 remaining subjects need less time in the adapted condition (C). In summary, the adaption of the stimulation can help to minimize measurement time and dropout of subjects.

For adapted trials, not fixating on the center of the screen resulted in an average delay of 2.3 s for each trial (± 2.9 s; High-Compliant: 0.26 s ± 0.3 s; Low-Compliant: 4.6 s ± 2.8 s). However, these delays result in 100% of valid (fixated) trials and extend the total measurement time on average for only about 1.3 min (± 1.3 min). To compare this delay with the timing of the non-adapted condition, we compared the necessary time to reach the same amount of valid trials in both conditions. This results in 54 s (± 19.2 s) less time needed in the adapted condition to reach the same amount of valid trials (up to 90%). Importantly, in the non-adapted condition none of the subjects reached more than 90% of valid trials overall (see Fig. 5).

#### Main effect of emotion

**Fig 6:**
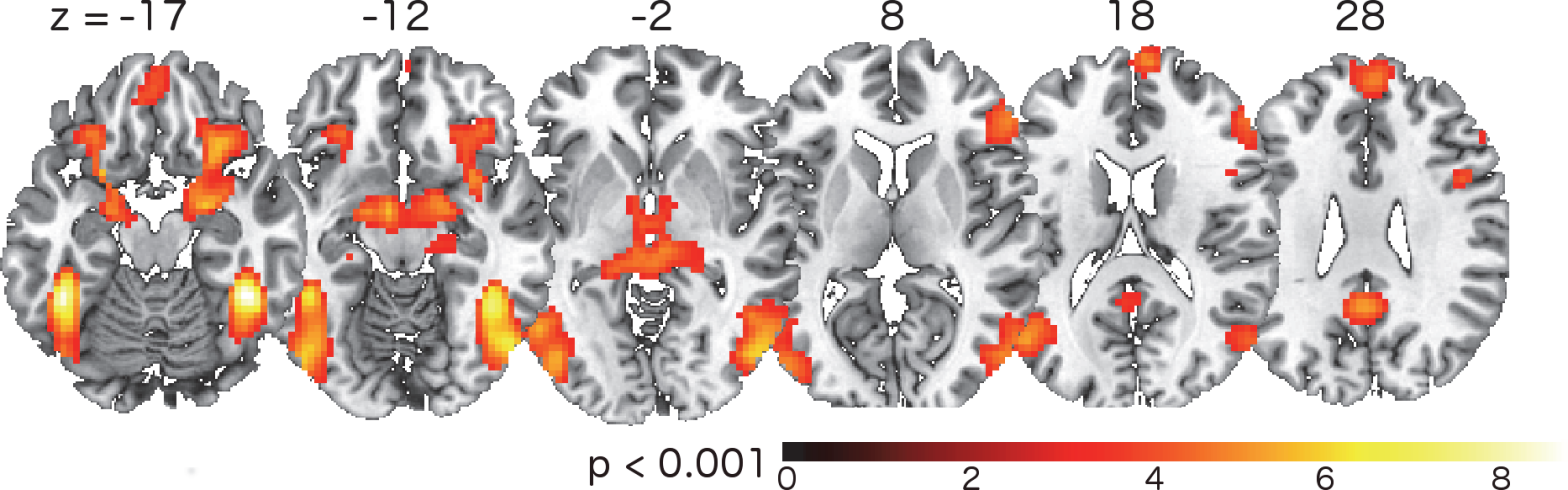
Main effect of emotion. All subjects’ t-map results for the second level analysis of ‘negative > neutral’ contrast.

In order to confirm a differential response to negative and neutral pictures, we first analyzed the main effect of emotion. Fig. 6 presents differences in brain activation between negative and neutral IAPS pictures. The activated brain regions are highly consistent with fMRI findings of previous studies using emotional scene stimuli [23], including the medial prefrontal cortex, bilateral limbic regions, bilateral pulvinar nuclei, and bilateral visual areas.

Table 1 summarizes in detail all significantly activated brain regions with their respective MNI coordinates and significance values.

**Table 1:** Activation network for the main effect of emotion (‘negative > neutral’ IAPS pictures; all subjects included).

**Region**		**MNI coordinates**				
		**x**	**y**	**z**	**t-Score** *	**z-Score** *
**Medial Prefrontal Cortex**		6	53	31	4.82	4.44
**Orbitofrontal Cortex**	R	30	23	-17	5.48	4.94
	L	-27	29	-14	5.03	4.6
**Inferior Frontal Gyrus**	R	45	8	31	4.39	4.09
**Insula**	R	33	8	-11	4.86	4.46
	L	-24	11	-17	5.52	4.97
**Amygdala**	R	21	-4	-14	5.65	5.07
	L	-18	-7	-14	5.04	4.61
**MDN Thalamus**	R	-3	-13	-2	4.23	3.96
**Pulvinar**	R	-15	-34	1	4.46	4.15
	L	15	-31	1	3.94	3.71
**Fusiform Gyrus**	R	42	-46	-17	8.39	6.86
	L	-42	-46	-20	8.29	6.81
**LateralOccipital Cortex**	R	51	-67	-8	6.84	5.9
	L	-45	-73	-11	5.71	5.11
**Middle Temporal Gyrus**	R	57	-67	16	5.03	4.6
	L	-48	-67	19	5.07	4.63
**Precuneus**		0	-52	28	5.06	4.62

*p < 0.001 (uncorr.), Abbreviations: L—left, R- right, MDN—Medial Dorsal Nuclei

#### Main effect of adaptation

**Fig 7:**
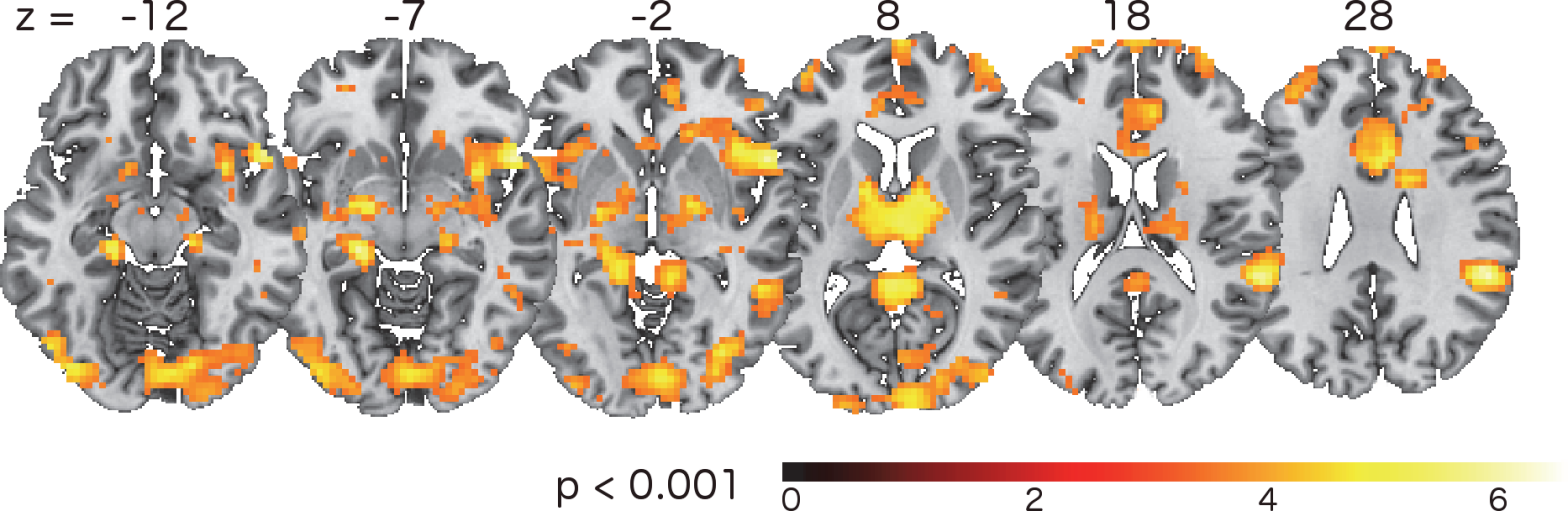
Main effect of adaptation. Low-Compliant’ t-map results for the second level analysis of ‘adapted-non > adapted’ contrast.

In order to examine whether the adaptation of the paradigm had an impact on neural activation, we contrasted adapted and non-adapted picture presentations for High- and Low-Compliant separately. As expected from their similar behavior in adapted and non-adapted trials, we found no significant neural alterations in fMRI signal for the High-Compliant group. In contrast, for Low-Compliant we found a significant increase of neural activation in the secondary visual cortex (brodman area 18), indicating an improved perception of the IAPS pictures and therefore underlining the benefits due to the adaptation (see Fig. 7). Furthermore, these adapted trials led to higher neural activation in several regions, which are known to underpin emotion processing: inferior frontal gyrus (IFG), hippocampus, BA 32, caudate, anterior cingulate, superior frontal gyrus, and pulvinar.

#### Effects of adaptation based on emotion related brain regions

**Fig 8:**
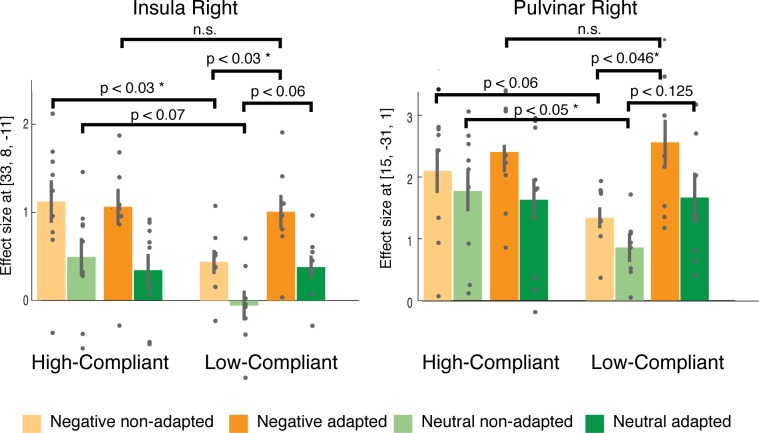
Exemplarily results for the increase of effect sizes due to adaption. In the ROI analysis for non-adapted vs. adapted picture presentations based on regions involved in emotional processing (main effect of emotion, Fig. 6) we found enhanced brain responses for adapted trials in Low-Compliant which, due to the adaptation, met the activation levels in High-Compliant. This picture shows this exemplarily for the right pulvinar and insula, the results are summarized in Table 2.

To illustrate the neural alterations due to the adaptation for the relevant brain regions of the emotional task, we directly compared the effect sizes for adapted and non-adapted trials in High- and Low-Compliant for relevant emotion-related regions. Therefore, we extracted the coordinates for the regions-of-interest (ROIs) from the main effect of emotion to compare the average signal from a 3 mm sphere between the adapted and non-adapted condition. Fig. 8 depicts the differences between Low- and High-Compliant for two representative examples, the right pulvinar and the insula cortex. In Low-Compliant, both adapted negative and neutral pictures led to significantly higher effect sizes compared to non-adapted trials. The adaptation of the picture presentation appeared to enhance the neural activation in these regions to the same activation level as observed in High-Compliant, again underlining the beneficial effect of adaptation.

Table 2 depicts all the brain regions that show enhanced effect sizes. In relation to our main hypothesis regarding the increase in statistical power when taking subjects’ gaze position into account, higher effect sizes were revealed by trials that were adapted to gaze position compared to non-adapted trials. This increase was found bilaterally in IFG, pulvinar nuclei, orbito frontal cortex (OFC), lateral occipital cortex (LOC), medial dorsal thalamic nuclei (MDN), and insula: brain regions implicated in emotion processing.

**Table 2:** Differences in effect sizes due to the adaptation of picture presentations in brain areas involved in emotional processing (ROIs taken from the main effect of emotions, see Fig. 6).

				**Low-Compliant**				**High-Compliant > Low-Compliant**			
**Region**	MNI Coordinates			Adapt > Non-AdaptNeg		Adapt > Non-AdaptNeut		Non-AdaptNeg		Non-AdaptNeut	
	x	y	z	p <	F	p <	F	p <	F	p <	F
**Insula R**	33	8	-11	0.03*	5.9	0.061	4.2	0.03*	5.7	0.07	3.9
**Pulvinar L**	-15	-34	1	0.07	3.8	0.104	3.0	0.02*	7.0	0.04*	4.9
**Pulvinar R**	15	-31	1	0.05*	4.8	0.125	2.7	0.058	4.2	0.05*	4.5
**OrbitofrontalCortex R**	30	23	-17	0.16	2.2	0.238	1.5	0.061	4.1	0.37	0.9
**OrbitofrontalCortex L**	-27	29	-14	0.36	0.9	0.14	2.5	0.04*	5.2	0.16	2.2
**Lateral OccipitalCortex L**	-43	-73	-11	0.19	1.9	0.25	1.4	0.001*	24.7	0.001*	19.4
**Lateral OccipitalCortex R**	51	-67	-8	0.22	1.7	0.175	2.0	0.01*	9.2	0.02*	7.5
**Inferior Frontal Gyrus**	33	17	-14	0.067	4.0	0.178	2.0	0.17	2.1	0.46	0.6
**MDN Thalamus L**	-3	-13	-2	0.242	1.5	0.03*	5.6	0.7	0.2	0.67	0.2
**MDN Thalamus R**	3	-13	-2	0.15	2.4	0.04*	5.0	0.7	0.2	0.85	0.04

For individual effect sizes see exemplarily Fig. 8.

Abbreviations: Neg—Negative; Neut—Neutral; Adapt—Adapted; Non-Adapt—non-adapted; R- right; L—left;

*—p < 0.05; F—effect size

#### Group comparison High- and Low-Compliant

Following the comparison of adapted and non-adapted trials, we also compared the two groups of High- and Low-Compliant for the adapted trials only. We did not find any of the previously mentioned brain regions in the contrast of all adapted trials between these two groups. This shows an increase in effect sizes for the Low-Compliant up to the level of High-Compliant for adapted trials. In other words, due to the adaptation there are no differences left and all subjects show more comparable results.

## Discussion

Our newly implemented software package BART offers a straightforward setup and a flexible, unified framework solution to adapt the stimulation according to various external parameters. As a usability example, we realized our first adapted paradigm, in which the subject’s off-center gaze position was monitored and demonstrated the benefits of using an online adapted paradigm for fMRI in a study with emotional pictures.

With this new technique, statistical power could clearly be increased in task-relevant regions, that is, involved in visual and emotional processing, for the Low-Compliant group (8 out of 17 subjects) up to the level of the High-Compliant. This was achieved by adapting the presentation of the pictures until the subject’s gaze position was directed towards the center of the screen. Additionally, this leads to a reduced measurement time and minimizes subject dropouts.

### Software package BART

Our newly developed software offers a highly flexible interface for various external devices and adapts the experimental fMRI paradigm to the measured behavioral or physiological parameters. Furthermore, it is built as a real-time fMRI system that could potentially be used in neurofeedback experiments. BART is clearly structured and divides logical units into separate modules—data input, real-time processing, stimulation, and handling of external devices. All parts of the software that are specific to an experimenter’s requirements are implemented as plugins. This allows for easy replacement of these parts or extension by new plugins. Of special importance in the present work are plugins for external devices measuring physiological or behavioral parameters.

In order to realize all these features in a unified framework, it was necessary to develop a generally applicable (i.e., abstract) concept. Here, this comprises: (1) a description of the adaptation within the realm of the configuration language EDL [15], which had to be extended by the new concepts described in section 2.1.; (2) an internal stimulation module, which can adapt a given paradigm according to the EDL description of external parameters; (3) a unified interface to connect different external devices to the stimulation module; and (4) the implementation of plugins to deal with the specific external device.

The stringent separation of these modules decouples the stimulation process and its adaptation from the acquisition and analysis of the influencing parameters, which are used for adaptation. This reduces the task of an adaptive paradigm setup from the complex four-step approach to the question of how to deal with a specific device and its parameters or a combination of different parameters.

To our knowledge, this is the first solution offering such a flexible interface for real-time analysis of data acquired from any modality (including fMRI) in order to manipulate the experimental protocol. Previously introduced software tools offered real-time physiological monitoring parameters and near real-time data processing [7,8,10] but no online adaptation. Another approach describes the use of an eye tracker to adapt the stimulation for more realistic experiments [9]. This is a very useful approach for more realistic social interactions but limited to eye movements, which describes a single use case setup. In contrast, the described abstract concept for BART allows for an integration of various other devices.

With BART, the task of an adaptive paradigm setup is reduced to implementing the details concerning the targeted parameter. Additionally, BART directly transfers all adapted trials into the design matrix for immediate data analysis.

All BART modules run in parallel on a single computer. This simplified setup increases the speed of data handling between the different software modules and significantly minimizes the error-proneness of the complex technical setup. The functionality has been tested under benchmark conditions, which proved accurate runtime behavior for the stimulation, adaptation, and transfer to the design matrix. In summary, BART can be used flexibly when researchers want to adapt a paradigm to an external measure or to improve the design of expensive studies in terms of measurement time or the number of available subjects (e.g., patients). Moreover, it can be used for real-time data analysis and therefore fits neurofeedback and biofeedback experiments. The sources of the software package are available online and further information regarding setup, usage, and plugin development is available upon request from the corresponding author.

In addition to the presented generalized approach for adaptive paradigms, the current work arose from the basic idea of biofeedback and neurofeedback in particular. These two concepts match each other for two reasons: Firstly, real-time fMRI needs trial-wise accuracy in order to reliably give information about brain states. Secondly, real-time fMRI results themselves can be used as a trigger for a stimulus presentation. So far, available software systems for real-time fMRI [13] do not contain sufficient flexible stimulation functionality.

### Usability experiment: Adaptive fMRI paradigm with emotional pictures

From the presented study we have clear evidence that subjects’ performance in gaze fixation is extremely poor on average. Even though we clearly instructed subjects to look at the fixation cross while no pictures were presented, some subjects did not fixate on it in up to 50% of the trials during presentation. Using an adaptive paradigm, we were able to raise the performance of these subjects up to the level of High-Compliant. This is shown by our results for the adapted picture presentation leading to significantly enhanced fMRI activation in relevant regions of the emotional task (main effect of emotion) and no differences left for adapted trials between High- and Low-Compliant.

First, we looked at results from standard analysis for data with emotional pictures (Fig. 6) that agree with the literature about emotion-processing brain regions [23]. It is also well known that visual awareness and attention highly influence the processing of the emotional stimuli [24]. Though visual fixation on a defined position does not guarantee correct perception of the content, it acts as an indicator for these properties. Our results from the comparison of adapted and non-adapted trials (Fig. 7) are consistent with emotional-processing pathways [25,26]: visual cortex (here, BA 18, LOC,), IFG, hippocampus, caudate, anterior cingulate, superior frontal gyrus, and precuneus. To quantify benefits for the emotional task from adaptation, we analyzed brain regions identified by main effect of emotion. This ROI analysis (Fig. 8) shows a significant increase in brain activation for adapted stimuli in Low- but not High-Compliant. Interestingly, we found an impact of adaptation on activation in the pulvinar nucleus. Recent models of emotional processing highlight the role of the pulvinar [25,27]. This region is known to be involved in attention functions [28] and was recently implicated in feature binding and working memory [29]. Although the current paradigm was not intended to examine the impact of attention on emotional processing, it is possible that the adaptation had an effect on deployment of attention to the pictures. In the future, the presented technique could be an appropriate tool to directly investigate the impact of attention on emotional processing. For example, an adaptive paradigm may be useful in this context based on previous work describing the relation between moment-to-moment fluctuations of brain responses and trial detection performance [30,31].

Regarding behavioral results, we found a significantly shorter response time for adapted trials. The valence and arousal ratings we obtained from the subjects in this study were in line with the available IAPS validation ratings, which verified our selection of representative subjects.

Although we found longer ITI times for the adapted trials in Low-Compliant, there is no evidence for an influence of this parameter on the brain activation results in our data (modeling the ITI in GLM gives quantitatively the same results; data not shown). But even taking time shifts due to the adaptation into account, less total experimental time is needed to reach the same amount of valid trials. Accordingly, by using an adaptive fMRI paradigm fewer trials are needed overall, leading to overall less measurement time. Additionally, the adaptation helps to minimize the number of dropouts of subjects. This is of special interest when number of available subjects, especially patients, is limited or post hoc correction for invalid trials, such as in real-time fMRI experiments, is simply not possible.

An alternative to waiting for subjects to fixate the center of the screen would have been to present the pictures at the current gaze position, which is possible with BART, thus overcoming this timing difference. This would be essential in more time-critical patient studies. In this work, we accepted this compromise to be as close as possible to gold standard where pictures are presented at the center of the screen.

In summary, the non-adapted presentation of pictures led to a higher variance in the neural activity due to differences in sensory stimulation and therefore includes an up to now non-quantified confound in conventional paradigms. By adaptation all trials are valid in the given context and the results presented here demonstrated that the level of effect sizes is maximized and stable among subjects. By less variance of noise in the data the overall statistical power is optimized due to the adaptation.

Next to emotional paradigms, the adaptation to a subject’s gaze position can be helpful in studies with disorders characterized by difficulties in fixating, such as autism or Huntington’s disease.

### Possibilities and limitations of adaptive paradigms

As shown in this work, the adaptation of fMRI paradigms can improve the statistical power of experiments. This can help to improve the reliability of fMRI in research and clinical applications, especially when the number of available subjects, the amount of stimulus material, or measurement time is strictly limited.

Adaptive paradigms can be a helpful tool to answer numerous questions. In addition to the example application demonstrated in this study, additional potential adaptive paradigms might be:

1. The adaptation of stimuli according to blood pressure parameters or heart rate variability can make sure that subjects are in a stressed or relaxed condition when stimuli are applied, e.g., in reappraisal studies.
2. Parameters from skin conductance or heart rate variability can be used to adapt the presentation of stimuli with individual emotional content in anxiety studies.
3. As an overall indicator of subjects’ vigilance state and attention, parameters from the EEG (e.g., mu rhythm or gamma band parameters) could be used to adapt the stimulation to increase the overall performance.
4. Parameters from the fMRI ‘BOLD state’ of a particular area, e.g., auditory, somatosensory cortex, could be used to adapt the presentation of modality-specific stimuli.

The last-mentioned approach is closely related to neurofeedback experiments because fMRI data has to be processed in real-time. But in contrast to a resulting learning signal, the results are used as a state marker to change the current paradigm.

Beside these advantages, there are also some limitations to adaptive paradigms. First, the external devices have to be compatible with an MR environment. Second, it is necessary that the external devices deliver a reliable measurement, both technically and physiologically. In the presented study we could not finish the measurements for some subjects because they had moved their head, which led to a signal loss for the eye tracker. Third, an important limitation of adaptive paradigms is the need to have a strong *a priori* hypothesis on how neural activation is influenced by the parameters used to adapt the paradigm. This gets even more complex for combined physiological parameters due to correlations between these parameters or for adaptations based on brain signals themselves from EEG or fMRI. Furthermore, there is a need for new analysis algorithms, as presented for example by Hinds et al., to analyze spontaneous fluctuations in brain activity [32]. When using fMRI results to adapt a paradigm accordingly, the signal from brain regions considered for adaptation purposes has to be independent from the post-hoc applied statistics. It is very important to avoid circular data manipulation and analysis [33]. As an example of independent influence, adaptive paradigms based on real-time fMRI analysis could investigate decision-making explicitly controlled by subjects’ emotional state, that is, asking for decisions when subjects are in an aroused or relaxed state, for example, as in a previous study by Brosch et al. [34].

In general, the most important aspect that has to be considered when using an adaptive paradigm is whether or not the variability of the measured behavioral or physiological parameter is important to address the question at hand. This would be the case, for example, when eye movements themselves influence the outcome of the investigated task or are constrained specifically by a disease. Ideally, an adaptation reduces the variability and helps to increase the statistical power and hence the validity of the results. In summary, an adaptation can increase the power of specific aspects of a study but reduces their variability.

## Conclusions

In conclusion, we present the newly implemented open source software package BART, which offers a flexible plugin-based approach for using different external modalities to trigger fMRI paradigms during runtime. Therefore, it is a well-suited tool to realize experiments with adaptive paradigms and it is fast to learn and operate. Additionally, it can be directly combined with real-time fMRI data processing.

We also present the benefits of the technique as a tool to improve the reliability of fMRI experiments. This technique yields possibilities to investigate novel scientific questions, such as the influence of EEG rhythms or other physiological parameters on hemodynamic brain responses.
